# Comment on Magner et al. Sulforaphane Treatment in Children with Autism: A Prospective Randomized Double-Blind Study. *Nutrients* 2023, *15*, 718

**DOI:** 10.3390/nu16050673

**Published:** 2024-02-28

**Authors:** Luke Curtis

**Affiliations:** Public Health Division, East Carolina University, Greenville, NC 27858, USA; lukecurtis1328@gmail.com; Tel.: +1-847-769-4768

I read your recent interesting double-blind study by Magner et al., which reported that treating 24 children with about 9 mg/day of phytochemical sulforaphane was associated with small and statistically insignificant improvements in social and autistic symptoms [1].

At least nine clinical studies have already reported that the consumption of cruciferous vegetables such as broccoli, cabbage, kale, radish, and bok choy, or the consumption of phytonutrients like sulforaphane, are associated with improved symptoms of patients with neurological conditions like autism or schizophrenia [2]. Earlier research has suggested that the oral consumption of several phytonutrients such as 1. sulforaphane, 2. resveratrol, 3. curcumin, and 4. naringenin may be helpful to treat autistic symptoms, and may be especially useful in a synergistic fashion when several of these phytonutrients are consumed at the same time [3]. Current research has found 10,000+ potentially helpful phytonutrients and at least seven major phytonutrient families which have a very broad range of health benefits including anti-inflammatory, anti-cancer, and neuroprotective properties [4]. The seven major phytonutrient families include 1. phenolic acids, 2. flavonoids, 3. tannins, 4. organosulfur compounds, 5. carotenoids, 6. anthocyanins, and 7. caffeine [4].

Vegetables and fruits are the main dietary source of a wide range of phytochemicals and other important nutrients such as vitamins A and C [4]. Autistic subjects often consume very low levels of vegetables and fruits, and hence, probably consume very low levels of beneficial phytonutrients. For example, one study of 48 autistic children reported a mean daily vegetable consumption of 0.55 daily servings as compared to 1.22 daily servings in 55 control children (*p* < 0.001) [5]. A prospective study of 5553 children aged 2 to 5 years old reported that 1581 children (28.5%) consumed an average of less than 0.5 servings of vegetables per day [6]!

A number of cases have reported on autistic children with severe deficiencies of vitamins A and C [7,8]. Such severe vitamin A and C deficiencies are rarely seen in non-impoverished populations and are probably due to the low vegetable and fruit consumptions in autistic children.

Much more clinical and research attention on phytochemicals and other nutritional factors are needed to optimally treat autistic patients. Since many autistic patients consume very low levels of fruits and vegetables, and since many phytonutrients may be helpful, treatment with single phytonutrients may not yield statistically significant improvements. The ideal nutritional treatment of autistic patients may involve a multifaceted strategy of a well-balanced diet rich in vegetables and fruits coupled with supplements of phytonutrients and other nutrients.
